# 毛细管电泳法高效筛选8-氧代鸟嘌呤DNA糖基化酶的核酸适配体

**DOI:** 10.3724/SP.J.1123.2020.12017

**Published:** 2021-07-08

**Authors:** Shimiao HAN, Liping ZHAO, Ge YANG, Feng QU

**Affiliations:** 北京理工大学生命学院, 北京 100081; School of Life Science, Beijing Institute of Technology, Beijing 100081, China; 北京理工大学生命学院, 北京 100081; School of Life Science, Beijing Institute of Technology, Beijing 100081, China; 北京理工大学生命学院, 北京 100081; School of Life Science, Beijing Institute of Technology, Beijing 100081, China; 北京理工大学生命学院, 北京 100081; School of Life Science, Beijing Institute of Technology, Beijing 100081, China

**Keywords:** 毛细管电泳, 核酸适配体, 8-氧代鸟嘌呤DNA糖基化酶, 竞争筛选, 多轮筛选, capillary electrophoresis (CE), aptamer, 8-oxoguanine DNA glycosylase, one-round pressure controllable selection, multi-round selection

## Abstract

8-氧代鸟嘌呤DNA糖基化酶(OGG1)是人体中重要的功能蛋白,在修复DNA氧化性损伤过程中起关键作用。氧化应激等引起的氧化损伤易导致炎症反应的发生,对OGG1的抑制可以一定程度上起到缓解作用;对癌细胞OGG1的抑制有望作为癌症治疗的新方法。目前的研究多集中于小分子对OGG1功能的影响和调控,而OGG1的适配体筛选尚未见报道。作为功能配体,适配体具有合成简单、高亲和力及高特异性等优点。该文筛选了OGG1的核酸适配体,结合毛细管电泳高效快速的优点建立了两种基于毛细管电泳-指数富集进化(CE-SELEX)技术的筛选方法:同步竞争法和多轮筛选法。同步竞争法利用单链结合蛋白(SSB)与核酸库中单链核酸的强结合能力,与目标蛋白OGG1组成竞争体系,并通过增加SSB浓度来增加竞争筛选压力,以去除与OGG1弱结合的核酸序列,一步筛选即可获得与OGG1强结合的核酸序列。多轮筛选法在相同孵育条件和电泳条件下,经3轮筛选获得OGG1的核酸适配体。比较两种筛选方法的筛选结果,筛选结果中频次最高的3条候选核酸适配体序列一致,其解离常数(*K*_D_)值在1.71~2.64 μmol/L之间。分子对接分析结果表明候选适配体1(Apt 1)可能与OGG1中具有修复氧化性损伤功能的活性口袋结合。通过对两种筛选方法的对比,证明同步竞争法更加快速高效,对其他蛋白核酸适配体筛选方法的选择具有一定的指导意义。得到的适配体有望用于OGG1功能调控,以抑制其修复功能。

## Abstract (en)

8-Oxoguanine DNA glycosylase (OGG1) is an important enzyme that plays a key role in oxidative DNA damage repair. OGG1 can specifically recognize and excise 8-oxoG (a product of oxidative damage found in double-stranded DNA) through base excision repair (BER). OGG1 is expressed in normal tissues, and in most tumor tissues. Oxidative cellular damage can produce an inflammatory reaction, alleviating some measure of constitutive OGG1 inhibition. OGG1 inhibition in cancer cells shows some promise as a new method of cancer treatment. Most current OGG1 research focuses on regulating OGG1 with targeted small molecules. To date, no aptamer screen for OGG1 has been reported. Aptamers are single-stranded DNA (ssDNA) or RNA oligonucleotides that can bind to a target with high affinity and specificity *in vitro*, that can be identified by systematic evolution of ligands by exponential enrichment (SELEX). Aptamers can be used as chemical ligands to regulate intermolecular interactions. In this study, a screen for aptamers with OGG1 affinity was performed for the first time. Capillary electrophoresis (CE) is a microanalytical technique that offers speed and high separation efficiency. In this work, two screening methods based on CE-SELEX technology were established: a one-round pressure controllable selection, and a multi-round selection. The most important criterion for successful one-round pressure controllable selection is to select a competitive target with a different CE migration time than that of the target of interest. We mixed OGG1 with a competitive target and a nucleic acid library for CE analysis. Two proteins competitively bind sequences in the library, forming independent complexes. The concentration of the competitive target is continuously increased until complexes with the target stop decreasing, indicating that the target and the ssDNA library have formed a stable complex. Complexes were collected for PCR amplification, purification, and high-throughput sequencing to obtain high affinity aptamers. This method greatly improves screening efficiency, and reduces non-specific binding to the target, which is helpful for obtaining aptamers with high affinity and specificity. One-round pressure controllable selection for high affinity OGG1 selective aptamers was performed using single strand binding protein (SSB) to competitively and tightly bind nucleic acids in the library. The competitive screening pressure was increased by increasing the SSB concentration to eliminate sequences with low affinity for OGG1 from the random oligonucleotide library. Nucleic acid sequences with high OGG1 affinity were obtainable in one step, and OGG1-ssDNA complexes were collected by creating a timed program on Beckman P/ACE MDQ capillary electrophoresis. Collection occurred from 2.2 to 2.8 min. Under identical incubation and electrophoresis conditions, multiple round selections were conducted by injecting samples of co-incubated nucleic acid library and target into the capillary. After separation under a high-voltage electric field, nucleic acid target complexes were collected, amplified by PCR, purified, and used as an enriched secondary library in the next round of screening. High affinity aptamers were generally obtained within three rounds. Comparing results of the two screening methods, the three candidate aptamer sequences found with the highest frequency were consistent, and displayed *K*_D_ values ranging from 1.71 to 2.64 μmol/L. Molecular docking analysis suggests that Apt 1 may bind to the OGG1 active pocket, which functions to repair oxidative damage. Comparison of the two screening methods indicates that one-round pressure controllable selection is more rapid and efficient, providing guidance for the design of other protein aptamer screening methods. The obtained aptamer is expected to be function effectively as an OGG1-mediated DNA repair inhibitor.

8-氧代鸟嘌呤DNA糖基化酶(OGG1)是修复DNA氧化性损伤的关键蛋白^[1,2]^,能够将DNA双链中的氧化损伤产物8-oxoG通过碱基切除修复途径(base-excision repair, BER)进行特异性识别和切除^[3]^。OGG1修复功能如果被抑制,将会导致正常细胞的DNA修复能力降低,DNA损伤蓄积,细胞凋亡增加^[4,5]^。但对于依赖DNA修复功能的癌细胞来说,OGG1蛋白修复功能的抑制则可以作为治疗癌症的新途径^[6]^。此外,OGG1也参与氧化应激等引发的炎症反应,在一些免疫调节疾病^[7,8]^中,过敏原会导致OGG1功能完好的上皮细胞产生更加严重的炎症反应^[9]^,因此,削弱OGG1的功能也能在一定程度上缓解炎症反应。

目前的研究工作多集中于小分子对OGG1功能的影响和调控研究,Donley等^[10]^筛选了数十种药物以抑制OGG1的修复功能,目前其药物的效用和特异性仍需验证;Visnes等^[11]^发现了一种OGG1的小分子抑制剂TH5487,能够减弱OGG1引起的炎症反应。对OGG1修复功能有抑制作用的配体的相关研究具有重要的理论意义和应用价值。核酸适配体是在体外通过指数富集配体进化技术(systematic evolution of ligands by exponential enrichment, SELEX)得到的与靶标以高亲和力和高特异性结合的单链DNA(ssDNA)或RNA。筛选OGG1蛋白的核酸适配体,可作为潜在的OGG1功能的调节因子,控制OGG1修复功能。目前,未见有OGG1蛋白的核酸适配体筛选及相关报道。

基于CE-SELEX筛选核酸适配体有多种方法^[12,13,14,15,16,17,18,19]^。其中利用毛细管区带电泳的多轮筛选方法是较为常用的筛选方法,通常需要3~4轮筛选可获得核酸适配体;本课题组最近发展了一轮同步竞争筛选方法^[20]^,通过在筛选过程中引入竞争靶标,并增加竞争靶标的浓度来增加筛选压力,同步提高筛选序列的亲和力和特异性。本文基于多轮筛选和一轮同步竞争筛选方法,筛选了OGG1的核酸适配体。筛选的示意图如图1所示,在竞争筛选中增加竞争靶标单链结合蛋白(SSB)的浓度就可以获得ssDNA与OGG1稳定结合的复合物;多轮筛选需要将ssDNA与OGG1的复合物收集后PCR形成Sub ssDNA以重复多轮筛选过程,收集亲和力不再提高的轮次的复合物,并利用高通量测序对两种复合物在PCR后获得的ssDNA进行分析。采用分子对接分析预测了核酸适配体Apt 1与OGG1的结合位点,发现该适配体可能与OGG1蛋白的活性口袋区域结合,有望成为该蛋白的调控因子。

**图 1:**
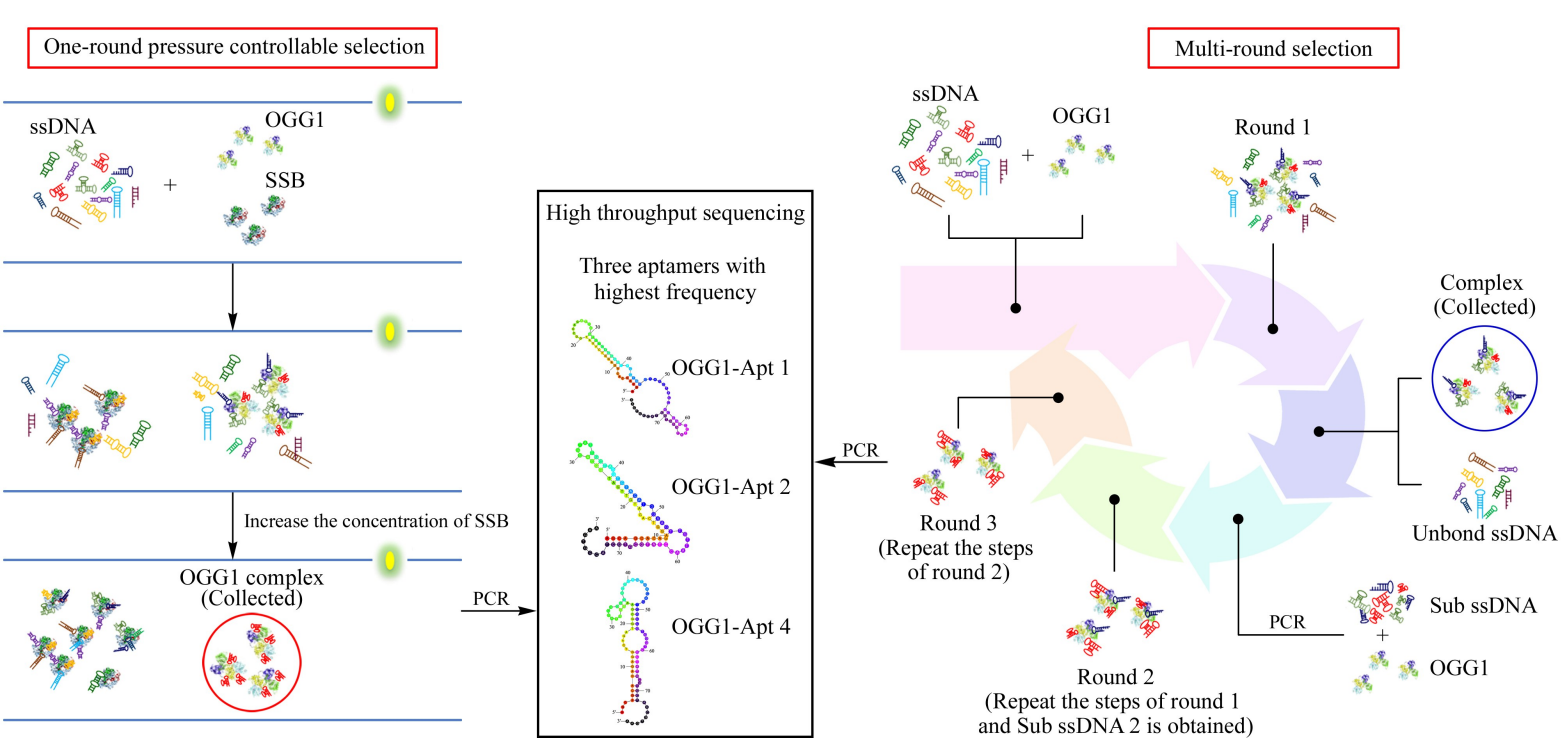
两种筛选方法原理示意图

## 1 实验部分

### 1.1 仪器与试剂

Beckman P/ACE MDQ毛细管电泳仪配荧光检测器(美国Beckman-coulter公司)、S1000^TM^ Thermal Cycler PCR仪(美国BIO-RAD公司)、Fresco21低温离心机(美国ThermoFisher Scientific公司)、PICO21台式高速离心机(美国ThermoFisher Scientific公司)、300 V/400 mA/75 W Power凝胶电泳仪(美国BIO-RAD公司)。

熔融石英毛细管:75 μm i.d.×50.2 cm (40 cm有效长度)(河北鑫诺光纤色谱有限公司)。硼酸、硼酸钠、氢氧化钠(分析纯,北京化工厂)。OGG1重组蛋白(美国ImmunoClone公司); 80 nt荧光素标记(FAM)标记的ssDNA核酸库:5'-FAM-AG-CAGCACAGAGGTCAGATG-40-CCTATGCGTGCT-ACCGTGAA-3'(两端为20 nt引物区固定序列,中间为40 nt的随机序列)。引物P1: 5'-AGCAGCACAGAGGTCAGATG-3',标记荧光的引物5'-FAM-P1:FAM-5'-AGCAGCACAGAGGTCAGATG-3'),引物P2:5'-TTCACGGTAGCACGCATAGG-3'(生工生物工程(上海)股份有限公司)。

### 1.2 溶液配制

毛细管电泳分离缓冲溶液:50 mmol/L硼酸-硼砂溶液(pH 7.8)。缓冲液需经0.22 μmol/L滤膜过滤后使用。ssDNA核酸库固体粉末,使用前需按照说明用超纯水溶解,配制母液。配制好的母液于94 ℃变性处理5 min,冷却至室温后取出备用。根据实验需要用孵育缓冲溶液稀释蛋白质和核酸溶液。

### 1.3 实验条件

1.3.1 毛细管电泳条件

电泳运行缓冲液为50 mmol/L pH 7.8硼酸-硼砂缓冲溶液;分离电压为15 kV;分离温度为25 ℃。进样压力为3.45 kPa,时间5 s。激光诱导荧光检测器(LIF)检测激发和发射波长分别为488 nm和520 nm;毛细管在样品进样前需依次使用0.1 mol/L NaOH、H_2_O和电泳缓冲液冲洗3 min。使用新毛细管时按照常规活化方法处理^[21]^。

1.3.2 PCR扩增条件及产物浓缩和纯化

详细步骤参考本课题组已报道的方法^[21]^,具体如下。

PCR扩增:将引物P1、P2、收集模板、2×Taq DNA聚合酶预混液、双蒸水混合,95 ℃预变性5 min,变性10 s, 59 ℃退火20 s, 72 ℃延伸30 s;循环20次后,72 ℃后延伸10 min。

PCR产物琼脂糖凝胶电泳:将PCR产物与DNA凝胶加样缓冲液混匀后依次注入进样孔中,90 V下电泳40 min,用凝胶成像分析仪拍照记录。

产物浓缩:PCR产物加入3 mol/L醋酸钠溶液及-20 ℃预冷的无水乙醇混匀,15000 r/min离心15 min;加入70%乙醇洗涤沉淀,弃去乙醇,沉淀干燥。

产物纯化:切下琼脂糖凝胶电泳上目标条带,转入1.5 mL离心管中,捣碎后加入ddH_2_O,振荡;加入Tris饱和纯苯酚,振荡后离心;将上清液移入新离心管,加入氯仿-异戊醇(24:1, v/v),振荡后离心;将上清液移入新离心管,加入无水乙醇、3 mol/L醋酸钠溶液,混匀后离心,弃去上清液,室温下风干沉淀物。

1.3.3 序列分析与亲和力表征

核酸序列高通量测序及核酸适配体合成均由生工生物公司完成,使用NUPACK分析序列二级结构。平衡解离常数*K*_D_计算参考NECCEEM方法^[22]^,利用公式(1)进行计算。


(1) $K_{\mathrm{D}}=\frac{P_{0}\left(1+\frac{A_{\mathrm{DNA}}}{A_{\mathrm{diss}}+A_{\mathrm{DNA} \cdot \mathrm{P}}}\right)-\mathrm{DNA}_{0}}{1+\frac{A_{\mathrm{diss}}+A_{\mathrm{DNA}} \cdot \mathrm{P}}{A_{\mathrm{DNA}}}}$

其中,*P*_0_和DNA_0_是OGG1和ssDNA的初始浓度,*A*_DNA_为游离ssDNA的峰面积,*A*_diss_为解离区峰面积,*A*_DNA·P_为OGG1-ssDNA复合物的峰面积。

1.3.4 分子对接

在RCSB PDB数据库中检索ID为2XHI的结构文件,由UCSF Chimera对其进行可视化并绘制出OGG1的3D结构。适配体的3D结构最初由RNA Composer软件绘制,再通过模式RNA网络服务器和MDWeb网络服务器转换为DNA 3D坐标。基于文献^[23]^和Uniprot数据库预测OGG1的结合残基,利用PatchDock对接程序对OGG1与适配体进行全局刚性对接以预测二者的结合模式。对接和模拟过程都优先选择默认参数,并通过FireDock方法^[24]^迅速消除PatchDock对接引入的空间冲突,完成侧链位置和相对蛋白质方向的细化和优化。消除空间位阻后,使用能量分析功能模块对对接模型根据界面能量得分进行排序。最终选取的对接模型其界面能量得分是最小化的范德华力、去溶剂化、静电、氢键、二硫键、*p*-堆积、脂类相互作用和旋转异构体偏好的加权组合。

## 2 结果与讨论

### 2.1 同步竞争法筛选OGG1核酸适配体

在竞争筛选方法中,竞争蛋白的选择原则主要有两个,一是可以选择不同的与核酸序列具有强结合能力的蛋白作为竞争蛋白以达到竞争筛选、提高筛选效率的目的;二是选择与靶标蛋白具有明显不同电泳迁移时间的竞争蛋白,以便于区别各自复合物峰,从而高效收集靶标蛋白与核酸序列的复合物。参考本课题组建立的同步竞争筛选法,并考虑SSB能够无序列选择性地与单链核酸以纳摩尔级别的*K*_D_结合^[20]^,且其复合物峰与OGG1复合物峰在电泳图谱中分离度较好,能够作为竞争蛋白结合与OGG1结合力弱的序列,因此选择SSB作为竞争靶标。将OGG1靶标、SSB反筛靶与ssDNA核酸库共同孵育。收集电泳图中的OGG1-ssDNA复合物部分,进行PCR扩增、纯化及高通量测序。SSB反筛靶的存在降低了靶标的非特异性结合,显著提高了筛选效率,有助于获得高亲和力、高特异性的适配体。

从图2可以看出,0.05 μmol/L的核酸库峰在6 min出现(见图2a)。加入1.0 μmol/L的反筛靶SSB后,游离核酸峰降低,同时在3.8 min处出现SSB-ssDNA的复合物峰(见图2b),由于该分离缓冲溶液条件下,加入的SSB蛋白羧基解离导致带负电的单链核酸本身带电性发生变化,荷质比变小,游离核酸峰迁移时间变快。加入5.0 μmol/L OGG1后,游离核酸库峰面积降低,在2.5 min处出现OGG1-ssDNA复合物峰(见图2c)。将5.0 μmol/L OGG1和1.0 μmol/L SSB及ssDNA库同时孵育,电泳图中出现两种复合物的峰(见图2d)。因SSB蛋白与ssDNA之间具有强结合作用,增大其浓度可以对OGG1与ssDNA的结合形成竞争,从而增加对OGG1适配体的筛选压力。在图2d~e中,SSB的浓度由1.0 μmol/L增加到2.0 μmol/L时,SSB-ssDNA复合物的峰面积增大,OGG1-ssDNA复合物的峰面积略有减小。增加SSB的浓度至4.0 μmol/L和8.0 μmol/L,结合加入不同浓度SSB后对OGG1-ssDNA复合物峰面积的统计(见图3), OGG1-ssDNA复合物的峰面积与2.0 μmol/L相比没有明显变化(见图2a~f),说明OGG1与ssDNA已形成稳定的复合物。通过在Beckman毛细管电泳仪上设置相应的时间程序,收集2.2~2.8 min区段的OGG1-ssDNA复合物,直接进行PCR扩增和纯化后,由生工生物科技有限公司进行高通量测序。

**图 2:**
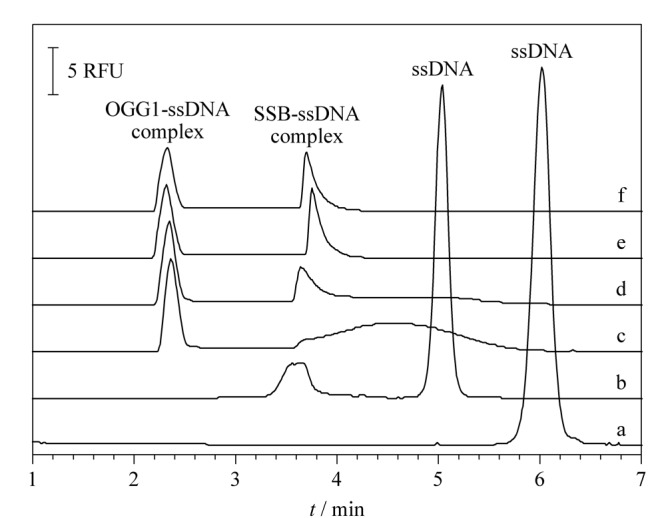
OGG1蛋白的竞争筛选

**图 3:**
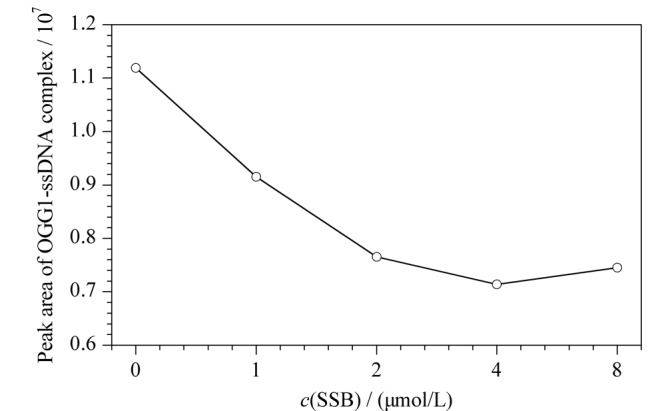
OGG1-ssDNA复合物峰面积变化

### 2.2 高通量测序及结果分析

剔除测序结果中读码区段错误及引物多聚体,选择频次最高的4条序列OGG1-Apt 1、2、4、6(见表1)。利用NUPACK、M-fold软件模拟序列二级结构及预测热力学参数,结果显示这些序列普遍形成茎环结构(见图4)。OGG1-Apt 1与OGG1-Apt 2在37 ℃时的Δ*G*较其他两条序列更低,说明这两条序列更易折叠成稳定的二级结构。

**表 1:** 4条候选适配体序列及其热力学参数

Aptamer	Sequence	ΔG/ (J/mol)	ΔH/ (J/mol)	ΔS/ (J/(K·mol))	T_m_/ ℃
OGG1-Apt 1	AGCAGCACAGAGGTCAGATGGCCACATTAGTCTCACCACTACCT GCGTACCTACCGCCGCCCTATGCGTGCTACCGTGAA	- 23571.67	- 510789.38	-1571.34	52
OGG1-Apt 2	AGCAGCACAGAGGTCAGATGTTGATGGCAGGTATTGCTAGGTCT ACATGGAACTTGTTAACCTATGCGTGCTACCGTGAA	- 26041.88	- 539678.29	-1655.96	52.7
OGG1-Apt 4	AGCAGCACAGAGGTCAGATGGCGAAGCGTACCGGCTACCCAGT GACAGTCGCCGTGGGTCCCTATGCGTGCTACCGTGAA	- 30312.42	- 458454.40	-1380.45	58.9
OGG1-Apt 6	AGCAGCACAGAGGTCAGATGCCGATCGTTGTTCCCGATCAAGAT GGGGAATCTCAGTCGCCCTATGCGTGCTACCGTGAA	- 28763.30	-417005.10	-1251.49	59.9

**图 4:**
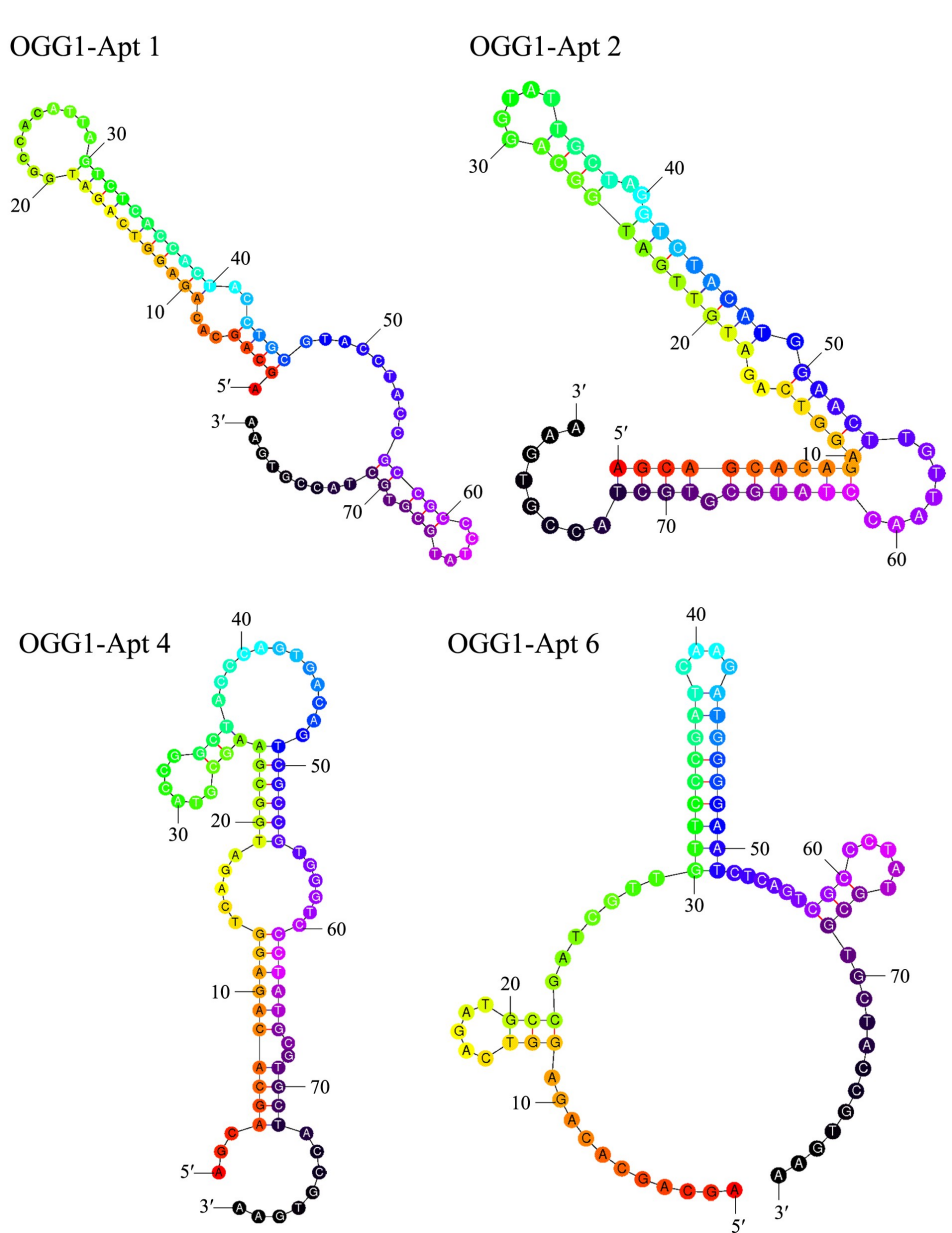
4条候选序列的二级结构

### 2.3 候选适配体的亲和力表征

通过CE方法对4条候选序列Apt 1、2、4、6进行亲和力表征并计算其*K*_D_值。图5中,0.2 μmol/L的Apt 1、2、4、6的电泳峰均在5.3 min处出现。加入5.0 μmol/L OGG1后,游离序列峰降低,在2.5 min处出现复合物峰(OGG1-Apt 1、2、4、6复合物),以及复合物峰与游离序列峰之间的解离区。通过对复合物峰、解离区、游离核酸峰三者峰面积的处理计算,OGG1-Apt 1、2、4、6的*K*_D_值分别为1.7±0.1 μmol/L、2.6±0.2 μmol/L、2.8±0.2 μmol/L、1.8±0.1 μmol/L(见图5)。

**图 5:**
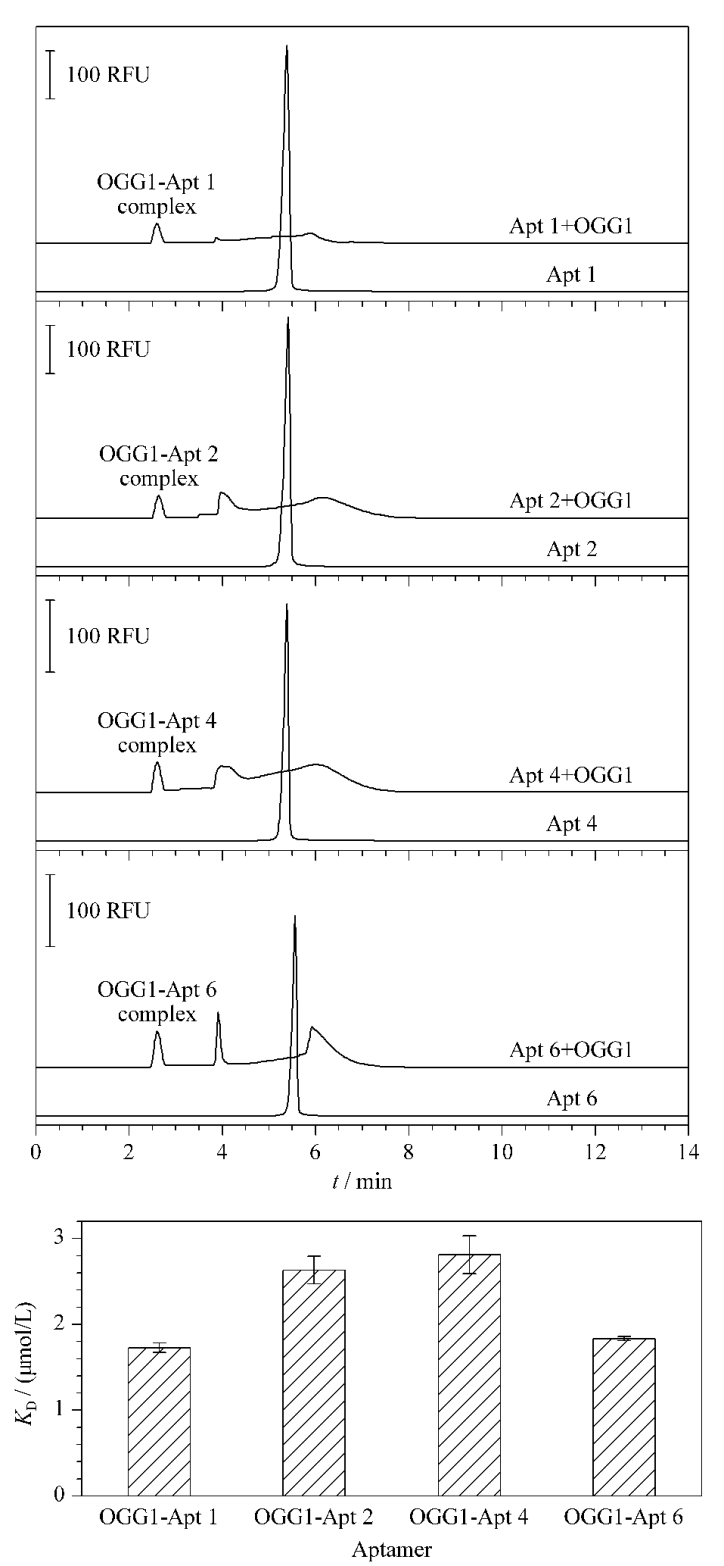
候选序列与OGG1结合的电泳图及*K*_D_值(*n*=3)

### 2.4 多轮筛选法筛选OGG1核酸适配体

多轮筛选法是将核酸库和靶标混合物孵育后进样,收集核酸-靶标复合物,并进行PCR扩增、核酸纯化,得到次级库(Sub-ssDNA),然后进行下一轮筛选。通常在4轮以内便能得到高亲和力的适配体。为了验证竞争筛选的结果,在相同的筛选条件下又采用多轮筛选法进行了筛选验证。

在第一轮筛选中,将0.2 μmol/L的ssDNA核酸库与5.0 μmol/L OGG1混合孵育15 min后,进行CE分析。在图6-Round 1中,游离核酸峰出现在5.1 min处,加入OGG1后,游离核酸峰面积降低,在2.5 min处出现OGG1-ssDNA复合物峰。设置相应的时间程序,对区间2.0~3.0 min的复合物进行收集,经对称PCR扩增、不对称PCR扩增及纯化过程,得到第一轮的次级库(Sub ssDNA1)以用于第二轮筛选。将第一轮得到的次级库与5.0 μmol/L OGG1混合孵育15 min后,进行CE分析,在图6-Round 2中,加入OGG1后游离Sub ssDNA1峰面积显著降低,在2.5 min处出现复合物峰,收集1.8~3.0 min区段复合物。经对称PCR扩增、不对称PCR扩增及纯化过程,得到第二轮的次级库(Sub ssDNA2)以用于第三轮筛选。保持筛选条件不变,将Sub ssDNA2与OGG1混合孵育进行第三轮筛选(图6-Round 3),收集1.9~3.0 min区段内复合物。随着筛选轮次的不断增加,与靶标弱结合的核酸序列不断被去除,使得亲和力更高的核酸序列保留下来。经三轮筛选后,分别计算蛋白与核酸初始库和次级库间的*K*_D_值:第一轮*K*_D_值为3.0 μmol/L,第二轮*K*_D_值为0.8 μmol/L,第三轮为1.0 μmol/L。实验结果表明,第二轮获得的核酸序列的亲和力较第一轮有一个数量级的提升,第三轮与第二轮所得核酸序列亲和力差别不大,并略弱于第二轮。因此,将第二轮获得的次级库经PCR扩增、纯化,送至生工生物科技有限公司进行高通量测序。

**图 6:**
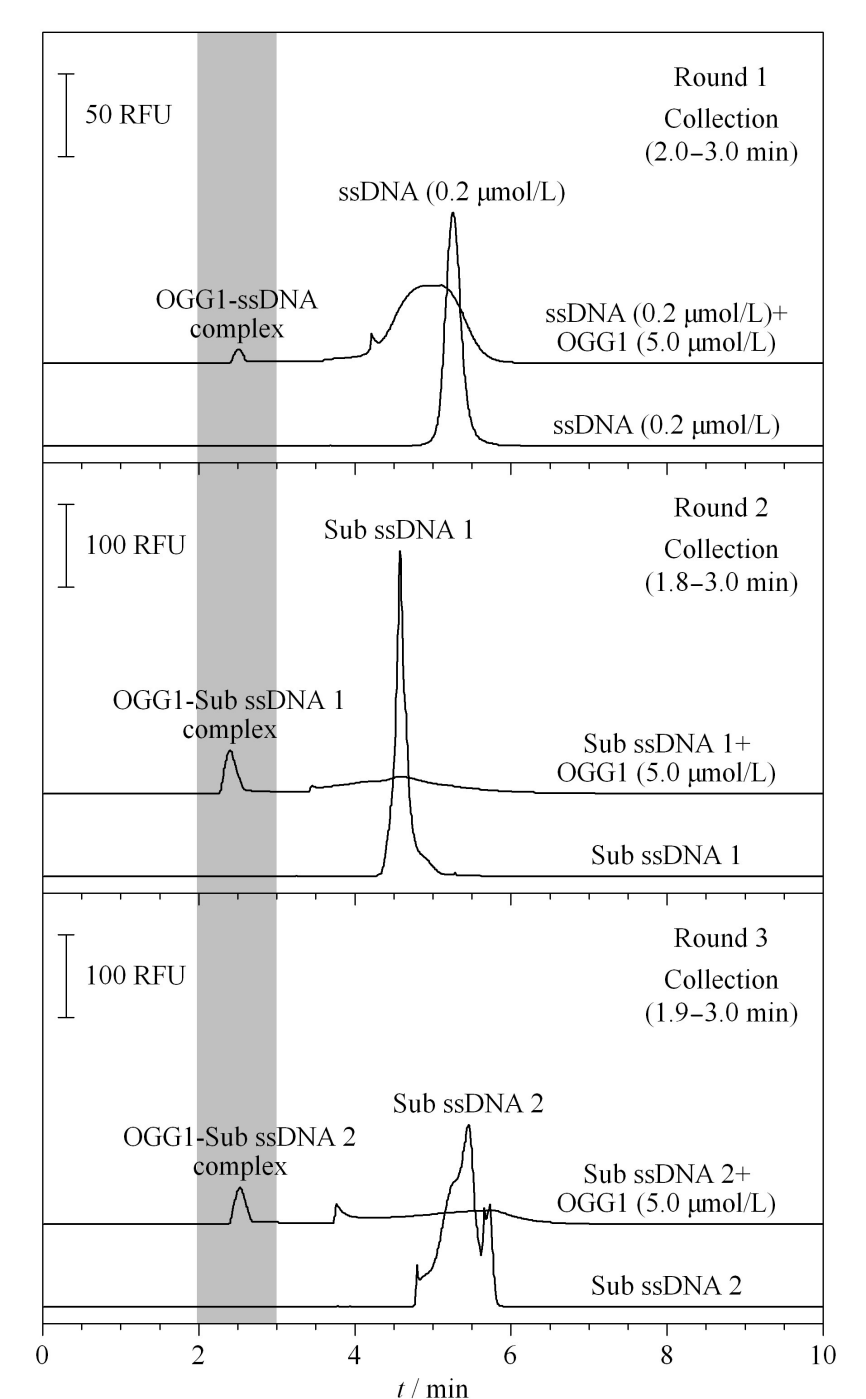
OGG1蛋白适配体3轮筛选的电泳图

### 2.5 高通量测序及结果分析

分析高通量测序结果,去除测序结果中读码区段错误及引物多聚体,选取其中最高频次的3条序列。将结果与竞争筛选法得到的序列进行对比(见表2),多轮筛选法筛选出的3条适配体序列与竞争法得到频次最高的3条适配体的序列相同。在多轮筛选中经过3轮筛选得到的序列频次最高达到1521次,而竞争筛选方法得到的适配体频次则较为均一(100次左右),多轮筛选过程是将可与靶标结合的核酸序列通过逐轮筛选富集,而同步竞争筛选法则是在共同孵育时,为靶标提供了一个强结合的反筛靶,通过竞争结合以更有效地获得高亲和力和高特异性的适配体序列。因此当存在合适的竞争蛋白的情况下,同步竞争筛选法仅通过一轮筛选即可获得亲和力和特异性更好的适配体,且使筛选过程加快,筛选时间和费用成本降低。

**表 2:** 多轮筛选与竞争筛选序列对比

Method	Sequence (5'-3')	Copy
Multi-round selection	AGCAGCACAGAGGTCAGATGGCCACATTAGTCTCACCACTACCTGCGTACCTACCGCCGCCC TATGCGTGCTACCGTGAA	1521
	AGCAGCACAGAGGTCAGATGTTGATGGCAGGTATTGCTAGGTCTACATGGAACTTGTTAACC TATGCGTGCTACCGTGAA	760
	AGCAGCACAGAGGTCAGATGGCGAAGCGTACCGGCTACCCAGTGACAGTCGCCGTGGGTCCC TATGCGTGCTACCGTGAA	36
One-round pressure controllable selection	AGCAGCACAGAGGTCAGATGGCCACATTAGTCTCACCACTACCTGCGTACCTACCGCCGCCC TATGCGTGCTACCGTGAA	132
	AGCAGCACAGAGGTCAGATGGCGAAGCGTACCGGCTACCCAGTGACAGTCGCCGTGGGTCCC TATGCGTGCTACCGTGAA	91
	AGCAGCACAGAGGTCAGATGTTGATGGCAGGTATTGCTAGGTCTACATGGAACTTGTTAACC TATGCGTGCTACCGTGAA	17

### 2.6 OGG1与Apt 1的分子对接分析

PatchDock是一种高效的基于几何形状互补增强的刚性对接方法。在PatchDock中输入两个分子的序列,并计算其中一个作为配体的分子相对于另一个作为受体的分子的三维变换。本次分析的两个分子是其中亲和力最高的OGG1-Apt 1及OGG1,根据给定的分子,PatchDock首先根据表面形状(凹陷,凸起或平整)将其表面分解成几个模块。然后,它执行几何散列算法以匹配具有平整形状的区块和具有凸起形状的模块,并生成一组有效的变换。将一组评分函数应用于每个候选变换来进行更深入的评估用以估计所获得的复合物的原子去溶剂化能量和形状互补性。最后,应用均方根偏差聚类(RMSD)将冗余的解决方案排除。在PatchDock成功对接并由FireDock进行细化后,选择10个原始的对接结果进行结合分析。

通过使用PyMol^[25]^进行对接结果分析,并基于全局自由能和结合残基选择最优的3个结果。OGG1在245~270位氨基酸残基处都具有螺旋-发夹-螺旋结构,该处是DNA的结合部位,同样也是酶的催化活性部位;Arg154和Arg204构成了识别8-oxoG-C碱基对的基础结构^[26]^; Asn149能够利用氢键将8-oxoG与OGG1形成的空隙填补;Cys249和Phe319之间会形成OGG1对8-oxoG的识别口袋,当氧化损伤发生产生的8-oxoG被修复时会插入该识别口袋;Gln315也对蛋白对底物的识别有用。分析对接结果显示从5'~3'端的50~80位的适配体残基与指定用于DNA和8-氧代鸟嘌呤结合的两个主要活性位点结合(见图7)。在OGG1适配体复合物中观察到不同类型的相互作用,即56个极性接触、26个范德华相互作用、39个氢键、6个疏水相互作用、14个离子相互作用和70个芳族相互作用。分子对接结果表明所筛选的适配体的50、51位胞嘧啶能与Arg154和Arg204形成氢键,且能够结合在OGG1蛋白的识别口袋上。

**图 7:**
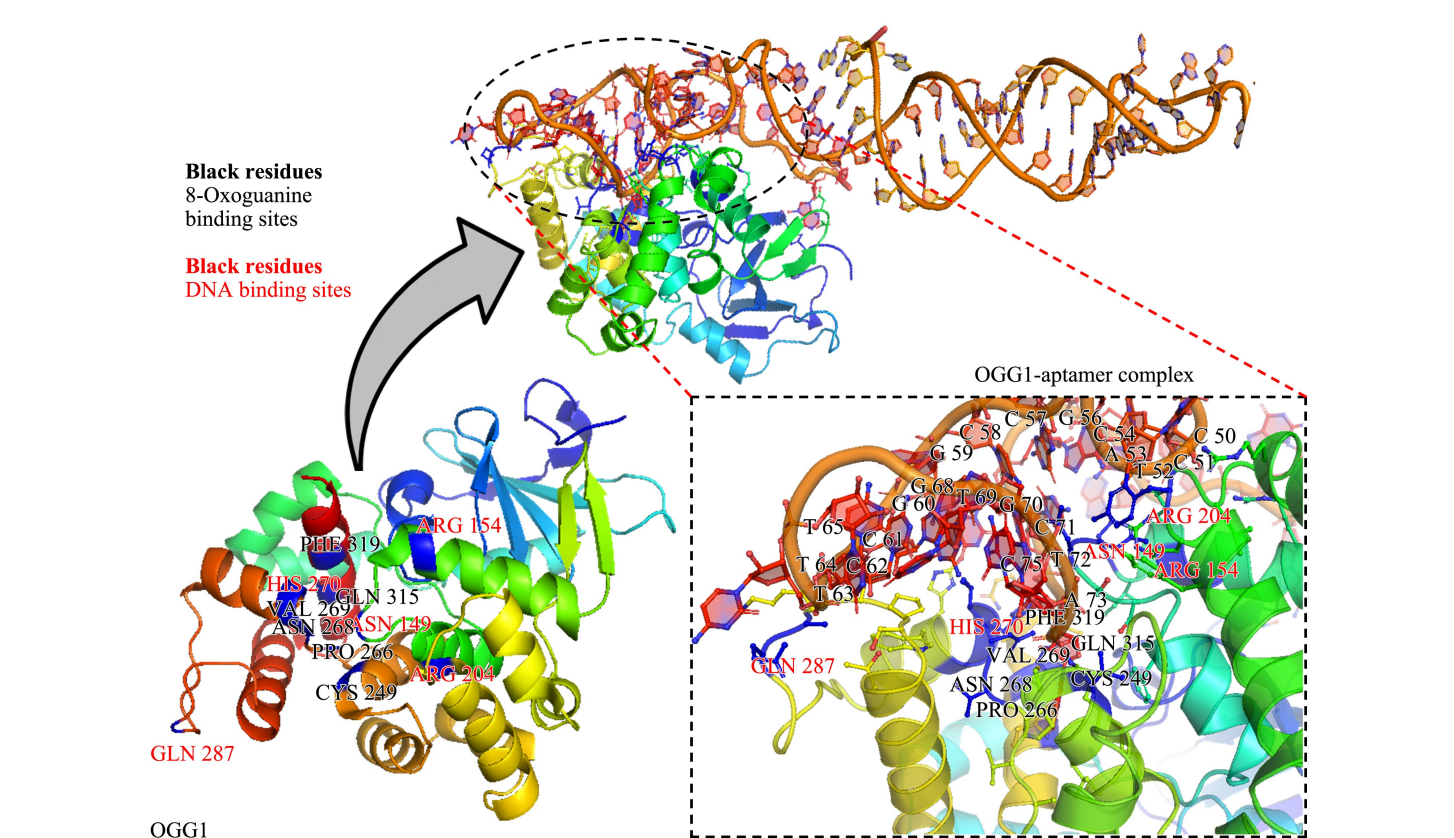
OGG1的活性位点的残基名称和标识符

## 3 结论

本文建立和比较了两种OGG1蛋白的核酸适配体筛选方法,即同步竞争筛选法及多轮筛选法。二者筛选到的3条适配体序列相同,同步竞争筛选法效率更高,可通过一轮筛选获得高亲和力、高特异性的适配体。分子对接分析表明,所筛选的Apt 1适配体可能结合于OGG1蛋白的活性口袋中,表明该适配体有望作为OGG1潜在的功能调控因子,抑制其修复作用。适配体对OGG1作用的相关研究正在进一步展开。
